# Association between metabolic syndrome and left ventricular geometric change including diastolic dysfunction

**DOI:** 10.1002/clc.23838

**Published:** 2022-05-03

**Authors:** Seung‐Jae Lee, Hyunah Kim, Byeong Kil Oh, Hyo‐In Choi, Ki‐Chul Sung, Jeonggyu Kang, Mi Yeon Lee, Jong‐Young Lee

**Affiliations:** ^1^ Division of Cardiology, Kangbuk Samsung Hospital Sungkyunkwan University School of Medicine Seoul Korea; ^2^ Center for Cohort Studies, Total Healthcare Center, Kangbuk Samsung Hospital Sungkyunkwan University School of Medicine Seoul Korea; ^3^ Division of Biostatistics, Department of R&D Management, Kangbuk Samsung Hospital Sungkyunkwan University School of Medicine Seoul Korea

**Keywords:** diastolic dysfunction, echocardiography, heart failure, left ventricle, metabolic syndrome, relaxation function, sex difference

## Abstract

**Background:**

We investigated the association between individual components of metabolic syndrome (MetS) and left ventricular (LV) geometric changes, including diastolic dysfunction, in a large cohort of healthy individuals.

**Methods:**

Overall, 148 461 adults who underwent echocardiography during a health‐screening program were enrolled. Geographic characteristics on echocardiography and several markers of LV relaxation function were identified according to individual MetS components. Univariate linear regression analysis and a multivariate regression model adjusted for factors known to influence LV relaxation function were conducted.

**Results:**

The prevalence of LV diastolic dysfunction (LVDD) was higher in the MetS group than in the non‐MetS group (0.56% vs. 0.27%, *p* < .001). In univariate and multivariate analyses, E/A ratio, e′ velocity, and left atrial volume index were significantly associated with each component of MetS and covariates (all *p* ≤ .001). In the age‐ and sex‐adjusted model, MetS was significantly associated with LVDD (odds ratio [95% confidence interval], 1.350 [1.103, 1.652]). However, subjects with more MetS components did not have a significantly higher risk of LVDD. As the analysis was stratified by sex, the multivariate regression model showed that MetS was significantly associated with LVDD only in men (1.3 [1.00, 1.68]) with higher risk in more MetS component (*p* for trend < .001). In particular, triglyceride (TG) and waist circumference (WC) among MetS components were significantly associated with LVDD in men.

**Conclusions:**

MetS was associated with the risk of LVDD, especially in men, with a dose‐dependent association between an increasing number of components of MetS and LVDD. TG and WC were independent risk factors for LVDD in men.

## 1 INTRODUCTION

Metabolic syndrome (MetS), once referred to as “Syndrome X” or "insulin resistance,” is a set of risk factors that are correlated with each other and occur together, increasing the incidence of atherosclerotic cardiovascular disease diseases such as coronary artery disease, stroke, diabetes, chronic kidney disease, and several cancers.^1^, ^2^, ^3^, ^4^ There are various diagnostic criteria in the definition of MetS by different organizations, but they generally include abdominal obesity, hypertension, hyperglycemia, hypertriglyceridaemia, and low high‐density lipoprotein cholesterol (HDL‐C).^5^ Left ventricular diastolic dysfunction (LVDD) refers to a condition in which the cardiac filling pressure increases due to impaired LV relaxation and abnormal stiffening of the LV chamber.^6^ Since LVDD is considered a pathophysiological abnormality in the development of heart failure with preserved ejection fraction (HFpEF), the presence of LVDD is also necessary for diagnosing HFpEF.^7^, ^8^ Therefore, LVDD is considered an important tool in clinical practice.

Although the pathophysiological mechanisms related to LVDD and MetS have not yet been clearly established, many epidemiological studies have reported a positive association between the two.^9^, ^10^, ^11^, ^12^, ^13^ However, most studies have a small sample size, which is likely to result in a bias or decrease in the representativeness of the entire population. Therefore, the purpose of this study was to evaluate whether MetS can affect LV geometric changes and relaxation function in relatively healthy young adults.

## 2 METHODS

### 2.1 Study population

**Figure 1:**
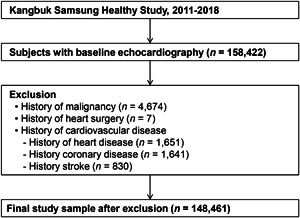
Flowchart summarizing the study population.

The study population consisted of individuals aged 18 years or older registered in the Kangbuk Samsung Health Study (KSHS). The KSHS is a cohort study of subjects who had a comprehensive health screening program at the Total Healthcare Center of the Kangbuk Samsung Hospital in Seoul and Suwon, Korea. The purpose of the comprehensive health screening program was to improve health status through the early detection of chronic diseases and associated risk factors. In Korea, the Industrial Safety and Health Law requires employees to undergo annual or biennial health examinations. Approximately 80% of the examinees are employees of various organizations and companies, and the rest are voluntarily registered in health screening programs. This study analyzed individuals who underwent echocardiography as a part of comprehensive health examination between January 2011 and December 2018 (Figure 1). To the 158 422 individuals for whom echocardiography and associated data were initially available, we applied the following exclusion criteria: history of malignancy (*n* = 4674), history of heart surgery (*n* = 7), history of heart disease (*n* = 1651), history of coronary disease (*n* = 1641), and history of stroke (*n* = 830). Finally, a total of 148 461 participants were eligible for inclusion in our study (102 416 men and 46 045 women; mean age, 40.3 ± 8.8 years). This study was approved by the Institutional Review Board of Kangbuk Samsung Hospital (IRB No: 2020‐03‐049). As anonymized and deidentified data were used for analysis, the need for informed consent was waived. Data supporting the findings of this study are available from the corresponding author upon request.

### 2.2 Clinical and laboratory measurements

All examinations were conducted at the Total Healthcare Center of the Kangbuk Samsung Hospital in Seoul and Suwon, according to a standardized protocol. Blood was drawn from participants after a fast of at least 10 h and analyzed at the Laboratory Medicine Department at Kangbuk Samsung Hospital, accredited by the Korean Society of Laboratory Medicine and the Korean Association of Quality Assurance for Clinical Laboratories.

Height, weight, waist circumference (WC), and body composition were measured by well‐trained nurses with participants wearing lightweight gowns. Body mass index (BMI) was calculated as weight in kilograms divided by height in meters squared. The WC was measured in the standing position, at the midpoint between the top of the anterior iliac crest and the lower border of the last palpable rib margin. Blood pressure and heart rate were measured using an automated oscillometric device (53000, Welch Allyn) by trained nurses while participants were in a sitting position with their arm supported at heart level after a 5‐min rest. Blood pressure and heart rate were measured three times in a row, and we used the average of the second and third readings for our analysis. The fat mass was measured using a multifrequency bioimpedance analyzer (Inbody 3.0, Inbody 720, Biospace Co.). As a marker of insulin resistance, the homeostasis model assessment of insulin resistance (HOMA‐IR) was calculated using equation^14^: HOMA‐IR = [fasting insulin (IU/ml) × fasting glucose (mmol/L)]/22.5.

Hypertension was defined as systolic blood pressure ≥140 mmHg, diastolic blood pressure ≥90 mmHg, selfreported history of hypertension, or current use of antihypertensive medications.^15^ Diabetes mellitus was defined based on the diagnostic criteria of the American Diabetes Association, a selfreported history of diabetes, or current use of antidiabetic medications.^16^ Dyslipidaemia was defined as a low‐density lipoprotein cholesterol (LDL‐C) level ≥ 160 mg/dl, HDL‐C level < 40 mg/dl, or triglyceride (TG) level ≥ 200 mg/dl, self‐reported history of dyslipidaemia, or history of medications for dyslipidaemia.^17^ Obesity was defined as a BMI ≥ 25 kg/m^2^ according to the Asia‐Pacific Region definition and the Korean Society for the Study of Obesity obesity guidelines.^17^, ^18^

### 2.3 Definition of MetS

MetS was defined according to the Joint Interim Statement of 2009.^19^ MetS was defined as the presence of any three or more of the following five criteria: (a) WC ≥ 90 cm in men or ≥80 cm in women; (b) TG level ≥ 150 mg/dl or drug treatment; (c) HDL‐C < 40 mg/dl in men or <50 mg/dl in women or drug treatment; (d) blood pressure ≥ 130/85 mmHg or antihypertensive medication; and (e) fasting glucose level ≥ 100 mg/dl or antidiabetic medication.

### 2.4 Echocardiographic data

Transthoracic echocardiography with a 4 MHz, sector‐type transducer probe (Vivid 7 and E9, General Electric) was performed by a trained sonographer following the guidelines.^6^, ^20^ Linear internal measurements of left ventricular end‐diastolic diameter (LVEDD), left ventricular end‐systolic diameter (LVESD), interventricular septum thickness (IVST), and posterior LV wall thickness (PWT) were acquired from the parasternal long‐axis view. LV mass was calculated through the linear method using the following cube formula: LV mass = 0.8 × [1.04 × (LVEDD + IVST + PWT)^3^ – LVEDD^3^] + 0.6 g. Left ventricular mass index (LVMI) was determined as LV mass/body surface area (g/m^2^). The left atrial (LA) size was obtained through the linear dimension method measuring the anteroposterior diameter of the LA. LV diastolic function was evaluated in the apical four‐chamber view using pulse‐wave Doppler with blood flow. Peak velocities of the early (E) and late (A) phases of mitral inflow and deceleration time of the E velocity were also measured. Peak early velocities of the early diastolic (e) and late diastolic (a′) phases were measured at the level of the septal mitral valve annulus using tissue Doppler imaging. We used the following four variables and abnormal cut‐off values to identify the LVDD: (a) septal e′ < 7 cm/s; (b) average E/e′ ratio > 14; (c) LA volume index > 34 ml/m^2^; (d) peak TR velocity > 2.8 m/s.^6^, ^20^ LVDD was defined if more than half of the available parameters met these cut‐off values.

### 2.5 Statistical analyses

Categorical variables are expressed as numbers (%) and compared using the *χ* ^2^ test. Continuous variables were expressed as mean (standard deviation) or median (interquartile range) according to their distribution. We used Student's *t*‐test or Mann—Whitney test to compare the two groups according to the presence/absence of MetS and LVDD, and analysis of variance (ANOVA) or Kruskal—Wallis tests to compare the four groups according to MetS and LV diastolic dysfunction. Linear regression and stepwise multiple regression analyses were performed to determine the association between diastolic measurement (E/A ratio, e′ velocity, and LA volume index) and potential variables, including clinical parameters, other echocardiographic parameters, and components of MetS. Odds ratios (ORs) and 95% confidence intervals (CIs) for LVDD according to MetS risk were estimated using multivariable logistic regression analysis. In our analyses, we used three models to adjust for confounding factors: model 1 was adjusted for age, sex, systolic blood pressure (SBP), diastolic blood pressure (DBP), TG, glucose, WC, BMI, HDL‐C, LDL‐C, and LVMI; model 2 was adjusted for age, sex, BMI, LDL, and LVMI; and model 3 was further adjusted by adding regular exercise, alcohol intake, and smoking status to the covariates of model 2. Associations between the number of MetS components and LVDD risk were evaluated using the same regression analyses. All *p* values were two‐tailed, and *p* < .05 was considered statically significant. All statistical analyses were conducted using STATA version 17.0 (StataCorp LP).

## 3 RESULTS

### 3.1 Baseline characteristics of the study population

In this study, 148 461 participants were included; 69% were male with a mean age of 40.3 ± 8.8 years. The prevalence of MetS was 16.8%. Table 1 shows the clinical, anthropometric, and echocardiographic characteristics of the study population, divided by comparing those with and without MetS. Participants with MetS were older (43.9 ± 9.5 vs. 39.6 ± 8.5 years), more obese (BMI, 27.3 ± 3.2 vs. 23.3 ± 3.4 kg/m^2^; WC, 93.1 ± 7.7 vs. 81.5 ± 8.5 cm) and more likely to be men (85.1% vs. 65.8%). They were more likely to be current or former smokers, more likely to drink, and less likely to exercise regularly. All metabolic values were significantly higher in the MetS group than in the MetS group. In addition, the proportions of current and former smokers and alcohol consumption were higher in the MetS group, and the proportion of those who exercised regularly was lower. Regarding echocardiographic parameters that reflect the cardiac structure, people with MetS showed statistically significant differences compared to those without MetS. In the MetS group, the ratio of LVDD was higher than in the group without MetS (0.56% vs. 0.27%, *p* < .001), and in detail, larger LA diameter (36.56 ± 4.13 mm vs. 32.79 ± 4.34 mm, *p* < .001), lower E/A ratio (1.18 ± 0.36 vs. 1.47 ± 0.43, *p* < .001), longer deceleration time (DecT) (191.23 ± 40.88 ms vs. 184.74 ± 36.88 ms, *p* < .001), and higher E/e′ ratio (8.05 ± 2.01 vs. 7.15 ± 1.62, *p* < .001) were found.

**Table 1:** Baseline characteristics of variables between individuals with and without MetS

	Total	MetS		
	(*n* = 148,461)	MetS (‐) (*n* = 123,578)	MetS (+) (*n* = 24,883)	*p*‐value
Age (years)	40.34 ± 8.84	39.61 ± 8.51	43.92 ± 9.54	<.001
Sex (male)	102 416 (68.99)	81 252 (65.75)	21 164 (85.05)	<.001
Hypertension	12 889 (8.68)	5923 (4.79)	6966 (28.00)	<.001
Diabetes	7068 (4.76)	2610 (2.11)	4458 (17.92)	<.001
Dyslipidaemia	45 939 (30.94)	27 494 (22.27)	18 445 (74.13)	<.001
Obesity	51 588 (34.75)	32 169 (26.03)	19 419 (78.04)	<.001
Systolic BP (mmHg)	111.07 ± 12.62	109.03 ± 11.61	121.17 ± 12.59	<.001
Diastolic BP (mmHg)	71.71 ± 9.9	70.14 ± 9.1	79.46 ± 10.07	<.001
Heart rate (bpm)	64.84 ± 9.1	64.23 ± 8.85	67.89 ± 9.69	<.001
BMI (kg/m^2^)	23.97 ± 3.35	23.29 ± 2.95	27.32 ± 3.17	<.001
Waist circumference (cm)	83.47 ± 9.44	81.49 ± 8.50	93.10 ± 7.70	<.001
Blood urea nitrogen (mg/dl)	12.75 ± 3.26	12.64 ± 3.20	13.29 ± 3.48	<.001
Creatinine (mg/dl)	0.86 ± 0.21	0.85 ± 0.20	0.91 ± 0.25	<.001
Total cholesterol (mg/dl)	194.68 ± 34.07	193.06 ± 32.94	202.73 ± 38.20	<.001
Triglyceride (mg/dl)	123.79 ± 85.20	106.84 ± 61.87	207.98 ± 125.62	<.001
HDL‐C (mg/dl)	57.1 ± 15.26	59.43 ± 14.82	45.51 ± 11.66	<.001
LDL‐C (mg/dl)	125.13 ± 32.05	123.40 ± 31.19	133.75 ± 34.75	<.001
Glucose (mg/dl)	96.19 ± 14.8	93.74 ± 10.95	108.32 ± 23.12	<.001
hsCRP (mg/dl)	0.11 ± 0.33	0.10 ± 0.33	0.15 ± 0.33	<.001
Insulin (_μ_U/ml)	6.86 ± 4.52	6.07 ± 3.58	10.78 ± 6.32	<.001
Fat mass (kg)	17.76 ± 6.09	16.63 ± 5.30	23.32 ± 6.64	<.001
HOMA‐IR	1.67 ± 1.31	1.42 ± 0.91	2.92 ± 2.05	<.001
Antihypertensive therapy	9593 (6.47)	3716 (3.01)	5877 (23.62)	<.001
Anti‐lipaemic therapy	5703 (3.85)	2587 (2.1)	3116 (12.52)	<.001
Diabetes mellitus therapy	3282 (2.21)	1222 (0.99)	2060 (8.28)	<.001
Smoking status				
Current smoker	28 284 (19.93)	21,334 (18.08)	6950 (29.11)	<.001
Former smoker	46 082 (32.48)	36 592 (31.01)	9490 (39.74)	<.001
Alcohol (g/day)	14.46 ± 21.70	13.14 ± 19.93	20.94 ± 28.00	<.001
Regular exercise (≥1 time per week)	58 737 (39.6)	49 129 (39.8)	9608 (38.6)	<.001
LVDD	471 (0.32)	332 (0.27)	139 (0.56)	<.001
Echocardiographic parameters				
IVSd (mm)	8.10 ± 1.28	7.95 ± 1.23	8.86 ± 1.22	<.001
LVPWd (mm)	8.00 ± 1.21	7.85 ± 1.17	8.74 ± 1.14	<.001
LVIDd (mm)	48.11 ± 4.07	47.96 ± 4.02	48.86 ± 4.24	<.001
LVIDs (mm)	30.28 ± 3.42	30.23 ± 3.39	30.53 ± 3.55	<.001
LA diameter (mm)	33.42 ± 4.53	32.79 ± 4.34	36.56 ± 4.13	<.001
E (m/s)	0.69 ± 0.15	0.70 ± 0.15	0.65 ± 0.14	<.001
A (m/s)	0.52 ± 0.13	0.50 ± 0.12	0.58 ± 0.14	<.001
E/A	1.42 ± 0.43	1.47 ± 0.43	1.18 ± 0.36	<.001
DecT (ms)	185.83 ± 37.66	184.74 ± 36.88	191.23 ± 40.88	<.001
e′ (m/s)	0.10 ± 0.02	0.10 ± 0.02	0.08 ± 0.02	<.001
E/e′	7.30 ± 1.73	7.15 ± 1.62	8.05 ± 2.01	<.001
LV mass index (g/m^2^)	129.54 ± 33.00	125.66 ± 31.70	148.39 ± 32.68	<.001
Ejection fraction (%)	66.63 ± 5.57	66.53 ± 5.52	67.13 ± 5.78	<.001

*Note*: Data are presented as *n* (%) or mean ± standard deviation. The blank fields were not significant.

Abbreviations: A, peak late diastolic transmitral flow; BMI, body mass index; BP, blood pressure; DecT, deceleration time; E, peak early diastolic transmitral flow; e′, early diastolic mitral annulus velocity; EF, ejection fraction; HDL‐C, high‐density lipoprotein cholesterol; HOMA‐IR, homeostasis model assessment of insulin resistance; hsCRP, high‐sensitivity C‐reactive protein; LA, left atrial; LDL‐C, low‐density lipoprotein cholesterol; LV, left ventricular; LVDD, left ventricular diastolic dysfunction; LVIDd, end‐diastolic left ventricular internal diameter; LVIDs, end‐systolic left ventricular internal diameter; LVPWd, end‐diastolic left ventricular posterior wall; MetS, metabolic syndrome.

Furthermore, when the entire population was divided according to the presence or absence of LVDD, the demographic and clinical characteristics showed significant differences (Table S1). The subjects with LVDD were older than the subjects without LVDD (46.55 ± 8.08 vs. 40.32 ± 8.84 years, *p* < .001), and had a higher proportion of each component of MetS. The echocardiographic parameters measured to evaluate diastolic dysfunction also showed significant differences between groups, excluding end‐diastolic left ventricular internal diameter, peak late diastolic transmitral flow (A), and left atrial volume index (LAVI). Table S2 shows the results of analyzing the total study population in more detail by dividing it into four groups according to the presence or absence of MetS and LVDD, and all variables showed significant differences. Table S3 shows the results of analysis of the entire population stratified by sex, and there were significant differences in demographic and clinical characteristics, except for age.

### 3.2 Univariate and multivariate regression analysis of LVDD

We used univariate and multivariate regression models to determine how each component of MetS, age, sex, and other factors affected the LVDD measures (Table 2). Univariate analysis demonstrated that all variables were statistically significantly related to E/A ratio, e′ velocity, and LAVI (*p* ≤ .007 for all). Similarly, the multivariate regression analysis demonstrated that all variables were independently related to the E/A ratio (*r* ^2^ = .375), e′ velocity (*r* ^2^ = .439), and LAVI (*r* ^2^ = .385) (*p* ≤ .005 for all).

**Table 2:** Correlation coefficients of univariate and multiple variable regression analyses of E/A ratio, e′ velocity and LA volume index for each component of the metabolic syndrome and covariates

Variable	E/A ratio				e′ velocity					LA volume index				
	Univariate		Multiple variable		Univariate			Multiple variable		Univariate			Multiple variable	
	*B (se)*	*p*‐value	*B (se)*	*p*‐value	*B (se)*		*p*‐value	*B (se)*	*p*‐value	*B (se)*		*p*‐value	*B (se)*	*p*‐value
Systolic BP	−0.010 (0.000)	<.001	0.002 (0.000)	<.001	−0.001 (0.000)		<.001	0.0001 (0.000)	<.001	0.200 (0.013)		<.001	0.136 (0.022)	<.001
Diastolic BP	−0.016 (0.000)	<.001	−0.011 (0.000)	<.001	−0.001 (0.000)		<.001	−0.001 (0.000)	<.001	0.165 (0.019)		<.001	−0.128 (0.028)	<.001
Triglyceride	−0.001 (0.000)	<.001	−0.00008 (0.000)	<.001	−0.00006 (0.000)		<.001	−0.00001 (0.000)	<.001	0.014 (0.002)		<.001		
Glucose	−0.006 (0.000)	<.001	−0.001 (0.000)	<.001	−0.0004 (0.000)		<.001	−0.00003 (0.000)	<.001	16.600 (1.238)		<.001		
Waist circumference	−0.014 (0.000)	<.001	−0.006 (0.000)	<.001	−0.001 (0.000)		<.001	−0.0002 (0.000)	<.001	0.294 (0.019)		<.001		
BMI	−0.036 (0.000)	<.001	−0.007 (0.001)	<.001	−0.002 (0.000)		<.001	−0.0004 (0.000)	<.001	0.840 (0.052)		<.001	0.492 (0.057)	<.001
HDL‐C	0.005 (0.000)	<.001	0.001 (0.000)	<.001	0.0003 (0.000)		<.001	0.00004 (0.000)	<.001	−0.063 (0.014)		<.001		
LDL‐C	−0.003 (0.000)	<.001	−0.001 (0.000)	<.001	−0.0001 (0.000)		<.001	−0.00004 (0.000)	<.001	0.018 (0.007)		<.007	−0.015 (0.005)	<.005
MetS (+) Group	−0.288 (0.003)	<.001			−0.017 (0.000)		<.001	−0.001 (0.000)	<.001	5.041 (0.465)		<.001		
Age	−0.025 (0.000)	<.001	−0.022 (0.000)	<.001	−0.001 (0.000)		<.001	−0.001 (0.000)	<.001	0.306 (0.02)		<.001	0.222 (0.019)	<.001
Sex	−0.132 (0.002)	<.001	0.034 (0.003)	<.001	−0.008 (0.000)		<.001	0.001 (0.000)	<.001	2.394 (0.447)		<.001		
LVMI	−0.01 (0.000)	<.001	0.001 (0.000)	<.001	−0.001 (0.000)		<.001	−0.000 (0.000)	<.001	0.302 (0.014)		<.001	0.141 (0.017)	<.001
Model adjusted *r* ^2^			.375					.439					.385	

*Note*: The blank fields were not significant. The β estimate represents the change in echocardiographic measures in patients with and without MetS.

Abbreviations: A, peak late diastolic transmitral flow; BMI, body mass index; BP, blood pressure; E, peak early diastolic transmitral flow; HDL‐C, high‐density lipoprotein cholesterol; LA, left atrial; LDL‐C, low‐density lipoprotein cholesterol; LV, left ventricular; LVMI, left ventricular mass index; MetS, metabolic syndrome.

### 3.3 Risk of LVDD according to MetS status

Table 3 shows the association between MetS and the risk of LVDD. In the univariate analysis, people with MetS had an increased risk of LVDD compared to those without MetS (OR, 2.085; 95% CI, 1.710–2.543; *p* < .001). Among those with MetS, the risk of LVDD increased with MetS components (3, 4, and 5 MetS risk factors vs. no MetS; OR, 1.790, 1.987, and 2.481; *p* ≤ .003 for all). In the analysis adjusting for age and sex, people with MetS remained a significant risk factor for LVDD (OR, 1.350; 95% CI, 1.103–1.652, *p* = .004). However, when analyzed by dividing people with MetS according to the number of components of MetS, the risk of LVDD seemed to increase as the number of components increased, but this was not statistically meaningful (3, 4, 5 and MetS risk factors vs. No MetS; OR, 1.216, 1.323 and 1.709; *p* ≥ .081 for all). In the multivariate analysis adjusted for age, sex, systolic BP, diastolic BP, TG, glucose, WC, BMI, HDL‐C, LDL‐C, and LVMI (model 1), people with MetS had a lower risk of LVDD than those without MetS, but there was no statistical significance, and the sub‐analysis showed similar results. Further analyses of model 2 (adjusted for age, sex, BMI, LDL, LVMI) and model 3 (adjusted for age, sex, BMI, LDL, LVMI, regular exercise, alcohol amount in grams, and current/former smoker) also showed that the risk of LVDD in the MetS group was not significant (models 2 and 3 vs. no MetS; OR, 1.081 and 1.094; *p* ≥ .495 for all). In addition, there was no statistical significance when analyzed according to the number of MetS components.

**Table 3:** Crude and adjusted odds ratios for the presence of any grade of diastolic dysfunction according to metabolic syndrome status

	Prevalence of LVDD	ORs (95% CIs)									
		Crude		Age‐ and sex‐adjusted		Model 1		Model 2		Model 3	
		OR (95% CI)	*p*‐value	OR (95% CI)	*p*‐value	OR (95% CI)	*p*‐value	OR (95% CI)	*p*‐value	OR (95% CI)	*p*‐value
No MetS (*n* = 123 578)	332 (0.30)	Reference		Reference		Reference		Reference		Reference	
Metabolic Syndrome (*n* = 24 883)	139 (0.60)	2.085 (1.710–2.543)	<.001	1.350 (1.103–1.652)	.004	0.888 (0.669–1.178)	.409	1.081 (0.843–1.386)	.541	1.094 (0.846–1.415)	.495
Three MetS risk factors (*n* = 16 293)	85 (0.50)	1.790 (1.415–2.266)	<.001	1.216 (0.959–1.543)	.106	0.913 (0.692–1.203)	.516	1.011 (0.771–1.326)	.935	1.012 (0.765–1.339)	.932
Four MetS risk factors (*n* = 7166)	43 (0.60)	1.987 (1.451–2.721)	<.001	1.323 (0.964–1.815)	.084	0.942 (0.645–1.376)	.758	1.107 (0.770–1.592)	.583	1.115 (0.767–1.621)	.570
Five MetS risk factors (*n* = 1424)	11 (0.80)	2.481 (1.361–4.521)	<.003	1.709 (0.935–3.123)	.081	1.084 (0.519–2.264)	.831	1.212 (0.591–2.486)	.600	1.287 (0.626–2.645)	.493

*Note*: Model 1 was adjusted for age, sex, systolic BP, diastolic BP, triglyceride, glucose, waist circumference, BMI, HDL‐C, LDL‐C, and LVMI. Model 2 was adjusted for age, sex, BMI, LDL‐C level, and LVMI. Model 3 was adjusted for age, sex, BMI, LDL‐C, LVMI, regular exercise, alcohol amount grams, current/former smoker.

Abbreviations: BMI, body mass index; BP, blood pressure; CI, confidence interval; HDL‐C, high‐density lipoprotein cholesterol; LDL‐C, low‐density lipoprotein cholesterol; LVDD, left ventricular diastolic dysfunction; LVMI, left ventricular mass index; MetS, metabolic syndrome; OR, odds ratio.

On the other hand, when people with LVDD were stratified by sex, it was confirmed that the risk of LVDD according to MetS was significantly associated, especially in men (OR [95% CI], 1.3 [1.00, 1.68] for men; 1.03 [0.39, 2.76] for women) (Table 4). In addition, there was a dose‐response relationship in the risk of LVDD according to the number of MetS components in men (3, 4, and 5 MetS risk factors vs. no MetS; OR [95% CI], 1.2 [0.89, 1.63], 1.5 [1.00, 2.24], and 2.1 [1.01, 4.38], respectively). As a result of analyzing the effect of each of the MetS criteria on the risk of LVDD, TG and WC had significantly increased the risk of LVDD after multivariate adjustment (OR, 1.3; 95% CI, 1.03–1.64 and 1.38; 1.02–1.85, respectively).

**Table 4:** Crude and adjusted odds ratios for the presence of any grade of diastolic dysfunction according to metabolic syndrome status

	Men			Women		
	Prevalence of LVDD, *n* (%)	Model 1	Model 2	Prevalence of LVDD, *n* (%)	Model 1	Model 2
No MetS	300 (69.44)/81 252	1 (reference)	1 (reference)	32 (82.05)/42 326	1 (reference)	1 (reference)
Total MetS	132 (30.56)/21 164	1.43 (1.14–1.79)	1.3 (1.0003–1.68)	7 (17.95)/3719	0.88 (0.35–2.21)	1.03 (0.39–2.76)
Three MetS risk factors	80 (21.05)/13 852	1.31 (1.01–1.71)	1.2 (0.89–1.63)	5 (13.51)/2441	0.97 (0.35–2.68)	1.11 (0.38–3.24)
Four MetS risk factors	41 (12.02)/6140	1.6 (1.12–2.29)	1.5 (1.002–2.24)	2 (5.88)/1026	0.81 (0.18–3.71)	0.9 (0.19–4.3)
Five MetS risk factors	11 (3.54)/1172	2.37 (1.26–4.46)	2.1 (1.01–4.38)	0 (0)/252	N/A	N/A
MetS components						
None	85 (19.68)/30 357	1 (reference)	1 (reference)	19 (48.72)/26 573	1 (reference)	1 (reference)
1 risk	116 (26.85)/28 797	1.2 (0.9–1.6)	1.15 (0.84–1.58)	7 (17.95)/10 834	0.45 (0.18–1.12)	0.57 (0.2–1.6)
2 risks	99 (22.92)/22 098	1.22 (0.9–1.66)	1.23 (0.87–1.72)	6 (15.38)/4919	0.46 (0.16–1.3)	0.41 (0.12–1.48)
3 risks	80 (18.52)/13 852	1.54 (1.1–2.16)	1.38 (0.94–2.02)	5 (12.82)/2441	0.54 (0.17–1.76)	0.66 (0.18–2.4)
4 risks	41 (9.49)/6140	1.8 (1.19–2.73)	1.66 (1.04–2.65)	2 (5.13)/1026	0.47 (0.09–2.41)	0.56 (0.1–3.19)
5 risks	11 (2.55)/1172	2.67 (1.38–5.17)	2.26 (1.05–4.88)	0 (0)/252	N/A	N/A
*p* for trend		<.001	<.001		.158	.310
MetS criteria						
Waist circumference	154 (35.65)/30 873	1.3 (0.999–1.69)	1.38 (1.02–1.85)*	10 (25.64)/6984	1.34 (0.51–3.53)	1.69 (0.58–4.9)
Triglyceride	233 (53.94)/43 083	1.32 (1.07–1.62)	1.3 (1.03–1.64)*	12 (30.77)/8200	0.98 (0.47–2.05)	1.25 (0.55–2.82)
HDL‐C	63 (14.58)/13 685	0.97 (0.73–1.28)	0.96 (0.7–1.32)	5 (12.82)/6664	0.59 (0.22–1.54)	0.4 (0.12–1.37)
Blood pressure	148 (34.26)/24 868	1.15 (0.93–1.43)	1.05 (0.82–1.34)	7 (17.95)/4604	0.53 (0.21–1.33)	0.66 (0.25–1.77)
Glucose	175 (40.51)/32 460	1.06 (0.87–1.3)	1.02 (0.81–1.28)	8 (20.51)/6907	0.77 (0.34–1.74)	0.64 (0.25–1.64)

*Note*: Model 1 was adjusted for age, obesity (BMI ≥ 25 kg/m²), and LDL‐C. Model 2 was adjusted for age, obesity (BMI ≥ 25 kg/m²), LDL‐C, LVMI, regular exercise, alcohol amount grams, current/former smoker.

Abbreviations: BMI, body mass index; BP, blood pressure; HDL‐C, high‐density lipoprotein cholesterol; LDL‐C, low‐density lipoprotein cholesterol; LV mass index, left ventricular mass index; LVDD, left ventricular diastolic dysfunction; MetS, metabolic syndrome.

*p < .001.

## 4 DISCUSSION

In this cross‐sectional cohort study, we showed that MetS was associated with LV geometric changes and diastolic dysfunction using echocardiographic measurements. The data were obtained from large samples of relatively young and healthy individuals. The main findings of the present study showed that (i) MetS increased the prevalence of LVDD; (ii) in age‐ and sex‐adjusted analysis, the risk of LVDD increased in the MetS group compared with the no MetS group, but the risk of LVDD did not tend to increase as the number of MetS components increased; (iii) after adjustment for multiple confounders, MetS showed a significant relationship with diastolic parameters, but it was difficult to find a significant association with the development of LVDD; and (iv) the correlation between MetS and LVDD was stronger in men than in women, and WC and TG were independent risk factors for LVDD. These findings suggest that MetS and each risk factor of MetS may cause a change in LV geometry as well as the diastolic parameters of the echocardiogram. In addition, it was suggested that not only does MetS have a sex difference in influencing diastolic dysfunction, but also that the components of MetS may not be affected with the same weight.

The exact pathophysiological mechanisms by which MetS induces the development of LVDD are unknown, but it is generally known that MetS is significantly associated with LVDD in several studies.^9^, ^10^, ^12^, ^21^ In two studies of subjects with normal LV function, MetS was associated with diastolic dysfunction regardless of LV hypertrophy,^9^, ^21^ and diastolic dysfunction occurred even in the pre‐MetS state.^9^ A Multi‐Ethnic Study of Atherosclerosis study using cardiac MRI for 1582 subjects showed that insulin resistance was associated with diastolic function, but MetS without type 2 DM could also develop diastolic dysfunction.^10^ A study of 684 Portuguese people showed a stepwise association between an increasing number of components of MetS and diastolic dysfunction.^12^ The mechanism by which the components of MetS induce LVDD is multifactorial and does not induce LVDD via different mechanisms. As a result, MetS is known to be a risk factor in the development of LVDD, but also to develop synergistically through the interaction of each component of MetS.^11^ Our results seem to be consistent with those of previous studies but showed some differences. The strength of our study and the distinction from other studies is that, through a large number of participants, MetS increased the risk of LVDD, especially in men, and that WC and TG could play an important role.

Waist circumference (abdominal obesity) is a well‐known cause of LVDD among the components of MetS, which can affect multiple metabolic and neurohormonal pathways due to accumulation of adipose tissue, causing abnormalities in the renin‐angiotensin system and myocardial oxidative stress.^22^ In addition, obesity causes cardiomyocyte apoptosis and cardiac structural remodeling due to an increase in lipotoxicity resulting from an increase in free fatty acid use, which can lead to diastolic dysfunction.^23^, ^24^, ^25^ Hyperglycemia causes an increase in oxidative stress by increasing fatty acid metabolism and reducing glucose metabolism,^26^ leading to contractile dysfunction,^27^ mitochondrial dysfunction,^28^ and endothelial dysfunction of cardiomyocytes.^29^ As TG levels increase, myocellular lipid accumulation increases, which is known to trigger lipoapoptosis and cause diastolic dysfunction.^30^ The combination of TG‐rich lipoprotein secretion and clearance impairment leads to abdominal obesity,^31^ and changes in TG levels could affect diastolic dysfunction by increasing the risk of diabetes.^32^ Low HDL levels not only do not sufficiently remove cellular lipids, but also cause arterial stiffness by not properly inducing NO synthesis, preventing apoptosis, and inducing angiogenesis, increasing myocardial cell hypertrophy and myocardial collagen, and eventually inducing diastolic dysfunction.^33^, ^34^ HTN increases LV after‐load and peripheral vascular resistance, causing LV structural remodeling.^35^, ^36^ This leads to myocardial fibrosis and LV hypertrophy, which increase the filling pressure, resulting in diastolic dysfunction.^37^

Unlike in other previous studies, it was difficult to conclude that the relationship between MetS and LVDD showed a significant trend after multivariate adjustment in this study. However, our study also confirmed that the MetS‐related variables were significantly associated with the diastolic parameters, as in other studies. Based on the consistent epidemiologic data that there was a sex difference in the prevalence of HFpEF,^38^ we were able to derive meaningful results by stratifying subjects with LVDD by sex. In addition, we divided the patients into four groups according to the presence or absence of MetS and LVDD, and further checked for regular exercise (Table S2). A recent study of 57 449 subjects suggested that physical activity may reduce the risk of impaired LV relaxation.^39^ Since the limitation of cross‐sectional studies is that the effects of differences in morbidity of each disease, changes in the condition of the disease due to drug use, and the causal relationship of exercise to the disease are difficult to elucidate, it is necessary to clarify our results through additional research.

## 5 STUDY LIMITATIONS

The results of this study should be interpreted in the context of several limitations. The main limitation of this study was that it was cross‐sectional and observational. Thus, our results might not only make it difficult to reach a conclusion about the causal relationship between MetS and LVDD, but might also be subject to unrecognized confounding factors or bias. Future research with a longitudinal design would provide better insights into the impact of the relationship between MetS and LVDD. Second, the majority of the study population was middle‐aged and living in urban areas, so selection bias may have arisen. It may be unreasonable to generalize our results to the entire population. On the other hand, since we conducted studies on young people who do not have multiple diseases that could act as a confounding factor, the effect of underlying diseases apart from MetS in this study would have been relatively small. Nevertheless, further studies are needed to confirm our findings among people of different age groups or other races/ethnicities. Third, we used a uniform cut‐off value without considering age as a criterion for diagnosing LVDD. In a study conducted with 2008 subjects, Miyoshi et al. suggested that as the diastolic parameters change with age in healthy people, the age‐specific criteria should be changed for an appropriate assessment of LV relaxation function.^40^ However, we targeted relatively young people with an average age of 40 years, so the elderly population is not large. Therefore, except for changes in LV diastolic parameters due to healthy aging, it is unlikely that the elderly participants who could be classified as normal belonged to the LVDD group and affected the study results.

## 6 CONCLUSIONS

In this study, we have shown that MetS is associated with the risk of LVDD in a dose‐dependent manner in the components of MetS, and there was a stronger association in men than in women. TG and WC were independent risk factors for LVDD in men. However, further studies are needed to clarify the specific mechanism and causal relationships between the components of MetS, sex differences, and LVDD.

## AUTHOR CONTRIBUTIONS

Jong‐Young Lee contributed to the conception and design of the study. Seung‐Jae Lee drafted the manuscript and edited the manuscript. Mi Yeon Lee contributed to the acquisition, analysis, and interpretation of data. Hyunah Kim, Byeong Kil Oh, Hyo‐In Choi, Jeonggyu Kang, and Ki‐Chul Sung contributed to the discussion. Jong‐Young Lee is the guarantor for this article. All the authors have read the manuscript and agree with the findings.

## CONFLICTS OF INTEREST

The authors declare no conflicts of interest.

## ETHICS STATEMENT

This study was approved by the Institutional Review Board of Kangbuk Samsung Hospital (IRB No. 2019‐05‐053).

## Supporting information

Supplementary Information

## Data Availability

Data supporting the findings of this study are available from the corresponding author upon request.

## References

[1] Grundy SM . Metabolic syndrome update. Trends Cardiovasc Med. 2016;26:364‐373. 26654259 10.1016/j.tcm.2015.10.004

[2] Reaven GM . Banting lecture 1988. Role of insulin resistance in human disease. Diabetes. 1988;37:1595‐1607. 3056758 10.2337/diab.37.12.1595

[3] Mottillo S , Filion KB , Genest J , et al. The metabolic syndrome and cardiovascular risk a systematic review and meta‐analysis. J Am Coll Cardiol. 2010;56:1113‐1132. 20863953 10.1016/j.jacc.2010.05.034

[4] Esposito K , Chiodini P , Colao A , Lenzi A , Giugliano D . Metabolic syndrome and risk of cancer: a systematic review and meta‐analysis. Diabetes Care. 2012;35:2402‐2411. 23093685 10.2337/dc12-0336 PMC3476894

[5] Silva V , Stanton KR , Grande AJ . Harmonizing the diagnosis of metabolic syndrome—focusing on abdominal obesity. Metab Syndr Relat Disord. 2013;11:102‐108. 23289486 10.1089/met.2012.0060

[6] Nagueh SF , Smiseth OA , Appleton CP , et al. Recommendations for the evaluation of left ventricular diastolic function by echocardiography: an update from the American Society of Echocardiography and the European Association of Cardiovascular Imaging. J Am Soc Echocardiogr. 2016;29:277‐314. 27037982 10.1016/j.echo.2016.01.011

[7] Kane GC , Karon BL , Mahoney DW , et al. Progression of left ventricular diastolic dysfunction and risk of heart failure. JAMA. 2011;306:856‐863. 21862747 10.1001/jama.2011.1201 PMC3269764

[8] Ponikowski P , Voors AA , Anker SD , et al. ESC Guidelines for the diagnosis and treatment of acute and chronic heart failure: the Task Force for the diagnosis and treatment of acute and chronic heart failure of the European Society of Cardiology (ESC) Developed with the special contribution of the Heart Failure Association (HFA) of the ESC. Eur Heart J. 2016;2016(37):2129‐2200. 10.1093/eurheartj/ehw128 27206819

[9] de las Fuentes L , Brown AL , Mathews SJ , et al. Metabolic syndrome is associated with abnormal left ventricular diastolic function independent of left ventricular mass. Eur Heart J. 2007;28:553‐559. 17311827 10.1093/eurheartj/ehl526

[10] Ladeiras‐Lopes R , Moreira HT , Bettencourt N , et al. Metabolic syndrome is associated with impaired diastolic function independently of MRI‐derived myocardial extracellular volume: the MESA study. Diabetes. 2018;67:1007‐1012. 29444891 10.2337/db17-1496 PMC5910005

[11] Hwang YC , Jee JH , Kang M , Rhee EJ , Sung J , Lee MK . Metabolic syndrome and insulin resistance are associated with abnormal left ventricular diastolic function and structure independent of blood pressure and fasting plasma glucose level. Int J Cardiol. 2012;159:107‐111. 21392830 10.1016/j.ijcard.2011.02.039

[12] Azevedo A , Bettencourt P , Almeida PB , et al. Increasing number of components of the metabolic syndrome and cardiac structural and functional abnormalities—cross‐sectional study of the general population. BMC Cardiovasc Disord. 2007;7:17. 17555566 10.1186/1471-2261-7-17 PMC1894986

[13] Gong HP , Tan HW , Fang NN , et al. Impaired left ventricular systolic and diastolic function in patients with metabolic syndrome as assessed by strain and strain rate imaging. Diabetes Res Clin Pract. 2009;83:300‐307. 19167773 10.1016/j.diabres.2008.10.018

[14] Matthews DR , Hosker JP , Rudenski AS , Naylor BA , Treacher DF , Turner RC . Homeostasis model assessment: insulin resistance and beta‐cell function from fasting plasma glucose and insulin concentrations in man. Diabetologia. 1985;28:412‐419. 3899825 10.1007/BF00280883

[15] Unger T , Borghi C , Charchar F , et al. International society of hypertension global hypertension practice guidelines. Hypertension. 2020;2020(75):1334‐1357. 10.1161/HYPERTENSIONAHA.120.15026 32370572

[16] American Diabetes Association . 2. Classification and diagnosis of diabetes: standards of medical care in diabetes‐2020. Diabetes Care. 2020;43:S14‐S31. 31862745 10.2337/dc20-S002

[17] Rhee EJ . Prevalence and current management of cardiovascular risk factors in Korean adults based on fact sheets. Endocrinol Metab (Seoul). 2020;35:85‐94. 32207267 10.3803/EnM.2020.35.1.85 PMC7090302

[18] WHO Expert Consultation . Appropriate body‐mass index for Asian populations and its implications for policy and intervention strategies. Lancet. 2004;363:157‐163. 14726171 10.1016/S0140-6736(03)15268-3

[19] Alberti KG , Eckel RH , Grundy SM , et al. Harmonizing the metabolic syndrome: a joint interim statement of the International Diabetes Federation Task Force on Epidemiology and Prevention; National Heart, Lung, and Blood Institute; American Heart Association; World Heart Federation; International Atherosclerosis Society; and International Association for the Study of Obesity. Circulation. 2009;120:1640‐1645. 19805654 10.1161/CIRCULATIONAHA.109.192644

[20] Lang RM , Badano LP , Mor‐Avi V , et al. Recommendations for cardiac chamber quantification by echocardiography in adults: an update from the American Society of Echocardiography and the European Association of Cardiovascular Imaging. Eur Heart J Cardiovasc Imaging. 2015;16:233‐270. 25712077 10.1093/ehjci/jev014

[21] Masugata H , Senda S , Goda F , et al. Left ventricular diastolic dysfunction as assessed by echocardiography in metabolic syndrome. Hypertens Res. 2006;29:897‐903. 17345790 10.1291/hypres.29.897

[22] Russo C , Jin Z , Homma S , et al. Effect of obesity and overweight on left ventricular diastolic function: a community‐based study in an elderly cohort. J Am Coll Cardiol. 2011;57:1368‐1374. 21414533 10.1016/j.jacc.2010.10.042 PMC3077126

[23] Rayner JJ , Banerjee R , Holloway CJ , et al. The relative contribution of metabolic and structural abnormalities to diastolic dysfunction in obesity. Int J Obes (Lond). 2018;42:441‐447. 28974742 10.1038/ijo.2017.239 PMC5880580

[24] Poirier P , Giles TD , Bray GA , et al. Obesity and cardiovascular disease: pathophysiology, evaluation, and effect of weight loss: an update of the 1997 American Heart Association Scientific Statement on Obesity and Heart Disease from the Obesity Committee of the Council on Nutrition, Physical Activity, and Metabolism. Circulation. 2006;113:898‐918. 16380542 10.1161/CIRCULATIONAHA.106.171016

[25] Cuspidi C , Rescaldani M , Tadic M , Sala C , Grassi G . Effects of bariatric surgery on cardiac structure and function: a systematic review and meta‐analysis. Am J Hypertens. 2014;27:146‐156. 24321879 10.1093/ajh/hpt215

[26] Li J , Shen X . Oxidative stress and adipokine levels were significantly correlated in diabetic patients with hyperglycemic crises. Diabetol Metab Syndr. 2019;11:13. 30774721 10.1186/s13098-019-0410-5 PMC6364461

[27] Singh RM , Waqar T , Howarth FC , Adeghate E , Bidasee K , Singh J . Hyperglycemia‐induced cardiac contractile dysfunction in the diabetic heart. Heart Fail Rev. 2018;23:37‐54. 29192360 10.1007/s10741-017-9663-y

[28] Verma SK , Garikipati VNS , Kishore R . Mitochondrial dysfunction and its impact on diabetic heart. Biochim Biophys Acta Mol Basis Dis. 2017;1863:1098‐1105. 27593695 10.1016/j.bbadis.2016.08.021 PMC5332436

[29] Bakker W , Eringa EC , Sipkema P , van Hinsbergh VW . Endothelial dysfunction and diabetes: roles of hyperglycemia, impaired insulin signaling and obesity. Cell Tissue Res. 2009;335:165‐189. 18941783 10.1007/s00441-008-0685-6

[30] de las Fuentes L , Waggoner AD , Brown AL , Dávila‐Román VG . Plasma triglyceride level is an independent predictor of altered left ventricular relaxation. J Am Soc Echocardiogr. 2005;18:1285‐1291. 16376756 10.1016/j.echo.2005.05.002

[31] Borén J , Watts GF , Adiels M , et al. Kinetic and related determinants of plasma triglyceride concentration in abdominal obesity: multicenter tracer kinetic study. Arterioscler Thromb Vasc Biol. 2015;35:2218‐2224. 26315407 10.1161/ATVBAHA.115.305614

[32] Tirosh A , Shai I , Bitzur R , et al. Changes in triglyceride levels over time and risk of type 2 diabetes in young men. Diabetes Care. 2008;31:2032‐2037. 18591400 10.2337/dc08-0825 PMC2551650

[33] Walter M . Interrelationships among HDL metabolism, aging, and atherosclerosis. Arterioscler Thromb Vasc Biol. 2009;29:1244‐1250. 19667114 10.1161/ATVBAHA.108.181438

[34] Mika M , Kanzaki H , Hasegawa T , et al. Arterial stiffening is a crucial factor for left ventricular diastolic dysfunction in a community‐based normotensive population. Int J Cardiol Hypertens. 2020;6:100038. 33447764 10.1016/j.ijchy.2020.100038 PMC7803042

[35] Pavlopoulos H , Grapsa J , Stefanadi E , et al. The evolution of diastolic dysfunction in the hypertensive disease. Eur J Echocardiogr. 2008;9:772‐778. 18490288 10.1093/ejechocard/jen145

[36] Kenchaiah S , Pfeffer MA . Cardiac remodeling in systemic hypertension. Med Clin North Am. 2004;88:115‐130. 14871054 10.1016/s0025-7125(03)00168-8

[37] Kai H , Kuwahara F , Tokuda K , Imaizumi T . Diastolic dysfunction in hypertensive hearts: roles of perivascular inflammation and reactive myocardial fibrosis. Hypertens Res. 2005;28:483‐490. 16231753 10.1291/hypres.28.483

[38] Beale AL , Meyer P , Marwick TH , Lam C , Kaye DM . Sex differences in cardiovascular pathophysiology: why women are overrepresented in heart failure with preserved ejection fraction. Circulation. 2018;138:198‐205. 29986961 10.1161/CIRCULATIONAHA.118.034271

[39] Ryu S , Chang Y , Kang J , et al. Physical activity and impaired left ventricular relaxation in middle aged adults. Sci Rep. 2018;8:12461. 30127508 10.1038/s41598-018-31018-z PMC6102302

[40] Miyoshi T , Addetia K , Citro R , et al. Left ventricular diastolic function in healthy adult individuals: results of the world alliance societies of echocardiography normal values study. J Am Soc Echocardiogr. 2020;33:1223‐1233. 32741597 10.1016/j.echo.2020.06.008

