# Supplementary material for: Association between metabolic syndrome and left ventricular geometric change including diastolic dysfunction
Source: Clin Cardiol. 2022 May 3;45(7):767–77. doi: 10.1002/clc.23838 (PMC9286337; doi:10.1002/clc.23838)
Supplement: Supplementary file 1 — Supplementary Information [file CLC-45-767-s001.docx]

| SUPPLEMENTARY TABLE 1. Baseline characteristics of variables between individuals with and without diastolic dysfunction | | | |
| --- | --- | --- | --- |
|  | **LV Diastolic Dysfunction** | | |
|  | **LVDD (-) (*n* = 147,990)** | **LVDD (+)  (*n* = 471)** | ***p* Value** |
| Age (years) | 40.32 ± 8.84 | 46.55 ± 8.08 | < .001 |
| Sex (male) | 101,984 (68.91) | 432 (91.72) | < .001 |
| Hypertension | 12,813 (8.66) | 76 (16.14) | < .001 |
| Diabetes | 7,019 (4.74) | 49 (10.4) | < .001 |
| Dyslipidaemia | 45,711 (30.92) | 228 (48.41) | < .001 |
| Obesity | 51,390 (34.73) | 198 (42.04) | .001 |
| Metabolic syndrome | 24,744 (16.7) | 139 (29.5) | < .001 |
| Systolic BP, (mmHg) | 111.05 ± 12.62 | 115.28 ± 11.73 | < .001 |
| Diastolic BP (mmHg) | 71.69 ± 9.89 | 77.69 ± 9.32 | < .001 |
| Heart rate (bpm) | 64.82 ± 9.09 | 70.24 ± 10.50 | < .001 |
| Body mass index (kg/m^2^) | 23.96 ± 3.35 | 25.03 ± 3.28 | < .001 |
| Waist circumference (cm) | 83.46 ± 9.44 | 87.66 ± 8.58 | < .001 |
| Blood urea nitrogen (mg/dL) | 12.75 ± 3.26 | 13.58 ± 3.29 | < .001 |
| Creatinine (mg/dL) | 0.86 ± 0.21 | 0.96 ± 0.16 | < .001 |
| Total cholesterol (mg/dL) | 194.65 ± 34.07 | 203.19 ± 33.64 | < .001 |
| Triglyceride (mg/dL) | 123.71 ± 85.16 | 148.68 ± 91.94 | < .001 |
| HDL cholesterol (mg/dL) | 57.11 ± 15.26 | 52.18 ± 12.89 | < .001 |
| LDL cholesterol (mg/dL) | 125.10 ± 32.04 | 135.50 ± 31.06 | < .001 |
| Glucose (mg/dL) | 96.17 ± 14.79 | 100.03 ± 15.86 | < .001 |
| hsCRP (mg/dL), median (Q1 ,Q3) | 0.05 (0.03-0.09) | 0.06 (0.03-0.12) | < .001 |
| Insulin (_μ_U/mL) | 6.85 ± 4.52 | 7.74 ± 4.70 | < .001 |
| Fat mass (kg) | 17.75 ± 6.09 | 18.82 ± 6.53 | < .001 |
| HOMA-IR, median (Q1, Q3) | 1.37 (0.91-2.05) | 1.69 (1.03-2.38) | < .001 |
| MetS | 24,744 (16.7) | 139 (29.5) | < .001 |
| Anti-hypertensive therapy | 9,541 (6.46) | 52 (11.04) | < .001 |
| Anti-lipaemic therapy | 5,672 (3.84) | 31 (6.58) | .002 |
| DM therapy | 3,263 (2.21) | 19 (4.03) | .007 |
| Smoking status |  |  | < .001 |
| Current smoker | 28,161 (19.91) | 123 (26.86) |  |
| Prior smoker | 45,874 (32.44) | 208 (45.41) |  |
| Regular exercise (≥1 time per week) | 58,532 (39.6) | 205 (43.5) | .015 |
| Echocardiographic parameters |  |  |  |
| IVSd (mm) | 8.10 ± 1.27 | 8.75 ± 1.39 | < .001 |
| LVPWd (mm) | 8.00 ± 1.21 | 8.51 ± 1.11 | < .001 |
| LVIDd (mm) | 48.11 ± 4.07 | 48.39 ± 4.26 | .131 |
| LVIDs (mm) | 30.28 ± 3.42 | 30.87 ± 3.32 | < .001 |
| LA diameter (mm) | 33.42 ± 4.53 | 34.46 ± 4.60 | < .001 |
| E (m/s) | 0.70 ± 0.15 | 0.42 ± 0.08 | < .001 |
| A (m/s) | 0.52 ± 0.13 | 0.52 ± 0.11 | .064 |
| E/A | 1.42 ± 0.43 | 0.82 ± 0.20 | < .001 |
| DecT (ms) | 185.75 ± 37.60 | 212.48 ± 45.45 | < .001 |
| E’ (m/s) | 0.10 ± 0.02 | 0.08 ± 0.04 | < .001 |
| E/e’ | 7.31 ± 1.73 | 5.15 ± 1.20 | < .001 |
| LV mass index (g/m^2^) | 129.50 ± 33.00 | 142.48 ± 31.63 | < .001 |
| LA volume index (ml/m^2^) | 23.44 ± 7.69 | 24.65 ± 6.68 | .753 |
| EF (%) | 66.63 ± 5.57 | 65.45 ± 5.79 | < .001 |
| Data are presented as n (%) or mean±SD.  Abbreviations: A, peak late diastolic transmitral flow; BMI, body mass index; BP, blood pressure; DecT, deceleration time; E, peak early diastolic transmitral flow; e’, early diastolic mitral annulus velocity; EF, ejection fraction; HDL-C, high-density lipoprotein cholesterol; HOMA-IR, homeostasis model assessment of insulin resistance; hsCRP, high-sensitivity C-reactive protein; LA, left atrial; LDL-C, low-density lipoprotein cholesterol; LV, left ventricular; LVDD, left ventricular diastolic dysfunction; LVIDd, end-diastolic left ventricular internal diameter; LVIDs, end-systolic left ventricular internal diameter; LVPWd, end-diastolic left ventricular posterior wall; MetS, metabolic syndrome.  The blank fields were not significant. | | | |

| SUPPLEMENTARY TABLE 2. Baseline characteristics of MetS and diastolic dysfunction in the entire cohort | | | | | |
| --- | --- | --- | --- | --- | --- |
|  | **MetS and diastolic dysfunction** | | | | |
|  | **MetS (-) and LVDD (-) (*n* = 123,246)** | **MetS (+) and LVDD (-) (*n* = 24,744)** | **MetS (-) and LVDD (+) (*n* = 332)** | **MetS (+) and LVDD (+) (*n* = 139)** | ***p* Value** |
| Age (years) | 39.6 ± 8.5 | 43.91 ± 9.55 | 46.48 ± 8.16 | 46.71 ± 7.93 | < .001 |
| Sex (male) | 80,952 (65.68) | 21,032 (85.00) | 300 (90.36) | 132 (94.96) | < .001 |
| Hypertension | 5,896 (4.78) | 6,917 (27.95) | 27 (8.13) | 49 (35.25) | < .001 |
| Diabetes | 2,592 (2.1) | 4,427 (17.89) | 18 (5.42) | 31 (22.3) | < .001 |
| Dyslipidaemia | 27,366 (22.23) | 18,345 (74.14) | 128 (38.55) | 100 (71.94) | < .001 |
| Obesity | 32,080 (26.03) | 19,310 (78.04) | 89 (26.81) | 109 (78.42) | < .001 |
| Systolic BP, (mmHg) | 109.02 ± 11.62 | 121.16 ± 12.59 | 112.19 ± 10.31 | 122.63 ± 11.67 | < .001 |
| Diastolic BP (mmHg) | 70.13 ± 9.09 | 79.44 ± 10.07 | 75.23 ± 8.17 | 83.53 ± 9.33 | < .001 |
| Heart rate (bpm) | 64.21 ± 8.84 | 67.87 ± 9.68 | 69.75 ± 10.32 | 71.42 ± 10.84 | < .001 |
| BMI (kg/m^2^) | 23.29 ± 2.95 | 27.32 ± 3.17 | 23.96 ± 2.39 | 27.57 ± 3.7 | < .001 |
| Waist circumference (cm) | 81.48 ± 8.51 | 93.09 ± 7.7 | 84.57 ± 6.69 | 94.93 ± 8.16 | < .001 |
| Blood urea nitrogen (mg/dL) | 12.64 ± 3.20 | 13.29 ± 3.48 | 13.73 ± 3.18 | 13.23 ± 3.52 | < .001 |
| Creatinine (mg/dL) | 0.85 ± 0.20 | 0.91 ± 0.25 | 0.96 ± 0.16 | 0.97 ± 0.16 | < .001 |
| Total cholesterol (mg/dL) | 193.03 ± 32.94 | 202.72 ± 38.21 | 202.42 ± 31.94 | 205.03 ± 37.47 | < .001 |
| Triglyceride (mg/dL) | 106.79 ± 61.87 | 207.99 ± 125.63 | 124.78 ± 59.84 | 205.76 ± 124.66 | < .001 |
| HDL cholesterol (mg/dL) | 59.45 ± 14.83 | 45.49 ± 11.65 | 54.08 ± 12.15 | 47.65 ± 13.51 | < .001 |
| LDL cholesterol (mg/dL) | 123.37 ± 31.18 | 133.75 ± 34.76 | 136.09 ± 29.76 | 134.08 ± 34.05 | < .001 |
| Glucose (mg/dL) | 93.74 ± 10.94 | 108.32 ± 23.14 | 96.38 ± 13.3 | 108.76 ± 17.98 | < .001 |
| hsCRP (mg/dL) | 0.10 ± 0.33 | 0.15 ± 0.33 | 0.14 ± 0.50 | 0.16 ± 0.16 | < .001 |
| Insulin (_μ_U/mL) | 6.07 ± 3.58 | 10.78 ± 6.32 | 6.37 ± 3.29 | 11.02 ± 5.85 | < .001 |
| Fat mass (kg) | 16.63 ± 5.3 | 23.32 ± 6.64 | 16.81 ± 4.72 | 23.65 ± 7.65 | < .001 |
| HOMA-IR, median (Q1, Q3) | 1.24 (0.84-1.78) | 2.45 (1.73-3.52) | 1.36 (0.9-1.95) | 2.47 (1.79-3.72) | < .001 |
| Antihypertensive therapy | 3,700 (3.01) | 5,841 (23.61) | 16 (4.82) | 36 (25.9) | < .001 |
| Anti-lipaemic therapy | 2,572 (2.09) | 3,100 (12.53) | 15 (4.52) | 16 (11.51) | < .001 |
| DM therapy | 1,212 (0.98) | 2,051 (8.29) | 10 (3.01) | 9 (6.47) | < .001 |
| Smoking |  |  |  |  | < .001 |
| Current smoker | 21,259 (18.06) | 6,902 (29.07) | 75 (23.36) | 48 (35.04) |  |
| Prior smoker | 36,444 (30.97) | 9,430 (39.72) | 148 (46.11) | 60 (43.8) |  |
| Regular exercise (≥1 time per week) | 48,978 (39.7) | 9,554 (38.6) | 151 (45.5) | 54 (38.8) | < .001 |
| Echocardiographic parameters |  |  |  |  |  |
| IVSd (mm) | 7.95 ± 1.23 | 8.85 ± 1.22 | 8.57 ± 1.44 | 9.18 ± 1.18 | < .001 |
| LVPWd (mm) | 7.85 ± 1.17 | 8.74 ± 1.14 | 8.34 ± 1.04 | 8.92 ± 1.15 | < .001 |
| LVIDd (mm) | 47.96 ± 4.02 | 48.86 ± 4.24 | 48.26 ± 4.04 | 48.71 ± 4.74 | < .001 |
| LVIDs (mm) | 30.23 ± 3.39 | 30.53 ± 3.55 | 30.90 ± 3.29 | 30.78 ± 3.42 | < .001 |
| LA diameter (mm) | 32.79 ± 4.34 | 36.56 ± 4.13 | 33.57 ± 4.46 | 36.59 ± 4.24 | < .001 |
| E (m/s) | 0.70 ± 0.15 | 0.65 ± 0.14 | 0.42 ± 0.08 | 0.41 ± 0.08 | < .001 |
| A (m/s) | 0.50 ± 0.12 | 0.58 ± 0.14 | 0.51 ± 0.10 | 0.55 ± 0.11 | < .001 |
| E/A | 1.47 ± 0.43 | 1.18 ± 0.35 | 0.85 ± 0.22 | 0.76 ± 0.16 | < .001 |
| DecT (ms) | 184.68 ± 36.83 | 191.08 ± 40.81 | 210.12 ± 45.64 | 218.08 ± 44.66 | < .001 |
| E’ (m/s) | 0.10 ± 0.02 | 0.08 ± 0.02 | 0.09 ± 0.04 | 0.08 ± 0.01 | < .001 |
| E/e’ | 7.16 ± 1.62 | 8.07 ± 2.00 | 5.11 ± 1.12 | 5.23 ± 1.35 | < .001 |
| LV mass index (g/m^2^) | 43.75 ± 9.72 | 50.53 ± 10.6 | 47.00 ± 8.80 | 51.22 ± 12.32 | < .001 |
| LA volume index (ml/ m^2^) | 22.17 ± 7.39 | 27.23 ± 7.30 | 22.03 ± 0.00 | 25.52 ± 7.90 | < .001 |
| EF (%), | 66.53 ± 5.52 | 67.14 ± 5.78 | 65.14 ± 5.94 | 66.21 ± 5.39 | < .001 |
| Data are presented as n (%) or mean ± SD.  Abbreviations: A, peak late diastolic transmitral flow; BMI, body mass index; BP, blood pressure; DecT, deceleration time; E, peak early diastolic transmitral flow; e’, early diastolic mitral annulus velocity; EF, ejection fraction; HDL-C, high-density lipoprotein cholesterol; HOMA-IR, homeostasis model assessment of insulin resistance; hsCRP, high-sensitivity C-reactive protein; LA, left atrial; LDL-C, low-density lipoprotein cholesterol; LV, left ventricular; LVDD, left ventricular diastolic dysfunction; LVIDd, end-diastolic left ventricular internal diameter; LVIDs, end-systolic left ventricular internal diameter; LVPWd, end-diastolic left ventricular posterior wall; MetS, metabolic syndrome.  The blank fields were not significant. | | | | | |

| SUPPLEMENTARY TABLE 3. Baseline characteristics of the study population stratified by sex. | | | |
| --- | --- | --- | --- |
|  | **Men**  **(*n* = 102,416, 68.99%)** | **Women**  **(*n* = 46,045, 31.01%)** | ***p* Value** |
| Age (years) | 40.31 ± 8.19 | 40.39 ± 10.14 | .144 |
| Hypertension | 10,407 (10.16) | 2,482 (5.39) | < .001 |
| Diabetes | 5,602 (5.47) | 1,466 (3.18) | < .001 |
| Dyslipidaemia | 38,604 (37.72) | 7,335 (15.95) | < .001 |
| Obesity | 44,101 (43.06) | 7,487 (16.26) | < .001 |
| Systolic BP, (mmHg) | 114.33 ± 11.37 | 103.79 ± 12.23 | < .001 |
| Diastolic BP (mmHg) | 74.29 ± 9.25 | 65.94 ± 8.79 | < .001 |
| Heart rate (bpm) | 64.64 ± 9.16 | 65.28 ± 8.96 | < .001 |
| BMI (kg/m^2^) | 24.79 ± 3.03 | 22.12 ± 3.28 | < .001 |
| Waist circumference (cm) | 86.54 ± 8.03 | 76.48 ± 8.66 | < .001 |
| Blood urea nitrogen (mg/dL) | 13.21 ± 3.17 | 11.72 ± 3.21 | < .001 |
| Creatinine (mg/dL) | 0.95 ± 0.19 | 0.67 ± 0.13 | < .001 |
| Total cholesterol (mg/dL) | 197.17 ± 33.87 | 189.13 ± 33.88 | < .001 |
| Triglyceride (mg/dL) | 139.26 ± 92.24, | 89.39 ± 52.57, | < .001 |
| HDL cholesterol (mg/dL) | 53.12 ± 13.20 | 65.95 ± 15.80 | < .001 |
| LDL cholesterol (mg/dL) | 129.62 ± 31.33 | 115.16 ± 31.37 | < .001 |
| Glucose (mg/dL) | 97.96 ± 15.31 | 92.24 ± 12.72 | < .001 |
| hsCRP (mg/dL) | 0.12 ± 0.35 | 0.09 ± 0.27 | < .001 |
| Insulin (_μ_U/mL) | 7.18 ± 4.57 | 6.13 ± 4.33 | < .001 |
| Fat mass (kg) | 17.85 ± 6.11 | 17.55 ± 6.02 | < .001 |
| HOMA-IR, median (Q1, Q3) | 1.47 (0.98-2.19) | 1.19 (0.79-1.75) | < .001 |
| Antihypertensive therapy | 7,479 (7.31) | 2,114 (4.6) | < .001 |
| Anti-lipaemic therapy | 4,120 (4.03) | 1,583 (3.44) | < .001 |
| DM therapy | 2,598 (2.54) | 684 (1.49) | < .001 |
| Smoke |  |  | < .001 |
| Current smoker | 27,576 (27.57) | 708 (1.69) |  |
| Prior smoker | 42,913 (42.91) | 3,169 (7.57) |  |
| Current/former smoker | 70,489 (68.83) | 3,877 (8.42) | < .001 |
| Alcohol amount grams (g/day) | 18.22 ± 23.71, | 5.23 ± 11.30 | < .001 |
| Regular exercise (≥1 time per week) | 45,837 (44.76) | 12,900 (28.02) | < .001 |
| LVDD | 432 (0.42) | 39 (0.08) | < .001 |
| Echocardiographic parameters |  |  |  |
| IVSd (mm) | 8.47 ± 1.18 | 7.29 ± 1.1 | < .001 |
| LVPWd (mm) | 8.36 ± 1.09 | 7.19 ± 1.06 | < .001 |
| LVIDd (mm) | 49.13 ± 3.84 | 45.85 ± 3.63 | < .001 |
| LVIDs (mm) | 30.99 ± 3.32 | 28.7 ± 3.07 | < .001 |
| LA diameter (mm) | 34.46 ± 4.29 | 31.11 ± 4.17 | < .001 |
| E (m/s) | 0.67 ± 0.13 | 0.76 ± 0.15 | < .001 |
| A (m/s) | 0.51 ± 0.12 | 0.54 ± 0.15 | < .001 |
| E/A | 1.38 ± 0.40 | 1.51 ± 0.48 | < .001 |
| DecT (ms) | 186.01 ± 37.32 | 185.43 ± 38.39 | .007 |
| e’ (m/s) | 0.10 ± 0.02 | 0.10 ± 0.02 | < .001 |
| E/e’ | 7.18 ± 1.65 | 7.60 ± 1.86 | < .001 |
| LV mass index (g/m^2^) | 141.03 ± 29.72 | 103.54 ± 24.07 | < .001 |
| EF (%) | 66.36 ± 5.58 | 67.22 ± 5.51 | < .001 |
| Data are presented as n (%) or mean ± SD.  Abbreviations: A, peak late diastolic transmitral flow; BMI, body mass index; BP, blood pressure; DecT, deceleration time; E, peak early diastolic transmitral flow; e’, early diastolic mitral annulus velocity; EF, ejection fraction; HDL-C, high-density lipoprotein cholesterol; HOMA-IR, homeostasis model assessment of insulin resistance; hsCRP, high-sensitivity C-reactive protein; LA, left atrial; LDL-C, low-density lipoprotein cholesterol; LV, left ventricular; LVDD, left ventricular diastolic dysfunction; LVIDd, end-diastolic left ventricular internal diameter; LVIDs, end-systolic left ventricular internal diameter; LVPWd, end-diastolic left ventricular posterior wall; MetS, metabolic syndrome.  The blank fields were not significant. | | | |
